# 慢性淋巴细胞白血病CD49d表达模式与分子遗传学和热点突变基因的相关性

**DOI:** 10.3760/cma.j.issn.0253-2727.2022.06.004

**Published:** 2022-06

**Authors:** 静 朱, 露 刘, 肖 陈, 芳 刘, 四书 赵, 慧敏 金, 海荣 仇, 纯 乔, 建勇 李, 雨洁 吴

**Affiliations:** 1 中国科学院大学附属肿瘤医院（浙江省肿瘤医院）检验科，中国科学院基础医学与肿瘤研究所，杭州 310022 Department of Clinical Laboratory, The Cancer Hospital of the University of Chinese Academy of Sciences (Zhejiang Cancer Hospital), Institute of Basic Medicine and Cancer (IBMC), Chinese Academy of Sciences, Hangzhou 310022, China; 2 南京医科大学第一附属医院，江苏省人民医院血液科，南京 210029 The First Affiliated Hospital of Nanjing Medical University, Department of Hematology, Jiangsu Province Hospital, Nanjing 210029, China

**Keywords:** CD49d, 分子遗传学, 基因突变, 流式细胞术, CD49d, Molecular genetics, Gene mutation, Flow cytometry

## Abstract

**目的:**

探索慢性淋巴细胞白血病患者CD49d表达模式与分子遗传学和热点突变基因的相关性。

**方法:**

流式细胞术检测CD49d表达模式，以单峰与双峰、阴性与阳性为分组依据。采用组合探针荧光原位杂交（FISH）进行分子遗传学分析，二代测序（NGS）进行基因突变检测。

**结果:**

CD49d阳性患者43例（23.89％），CD49d阴性患者137例（76.11％），CD49d单峰表达患者96例（53.33％），CD49d双峰表达患者84例（46.67％）。与CD49d阴性组患者相比，CD49d阳性组患者Rai分期高（*P*＝0.048），脾脏增大比例高（*P*＝0.030）。与CD49d单峰患者相比，CD49d双峰患者脾脏增大比例高（*P*＝0.009）。CD49d双峰阴性组11q22−发生率显著高于单峰阴性组（24.29％对10.45％，*P*＝0.043）。CD49d单峰组+12发生率高于双峰组（16.67％对5.95％，*P*＝0.035），单峰阳性组+12发生率高于双峰阴性组（17.24％对4.29％，*P*＝0.045），单峰阴性组+12发生率高于双峰阴性组（16.42％对4.29％，*P*＝0.024）。CD49d阳性组BIRC3突变率高于CD49d阴性组（11.63％对2.92％，*P*＝0.037）。

**结论:**

CD49d与11q22−、+12和BIRC3基因突变有显著的相关性，CD49d呈双峰表达与预后较差指标相关。

## Abstract (en)

**Objective:**

To explore the correlation of CD49d expression patterns with molecular genetics and hotspot gene mutants in patients with chronic lymphocytic leukemia.

**Methods:**

The expression of CD49d was detected by flow cytometry and grouped into homogeneous, bimodal, negative and positive expression. Panel fluorescence in situ hybridization (FISH) was used for molecular genetics analysis and next-generation sequencing (NGS) was conducted for gene mutation detection.

**Results:**

There were 43 patients (23.89％) with positive CD49d expression, 137 patients (76.11％) with negative CD49d expression, 96 patients (53.33％) with homogeneous CD49d expression and 84 patients (46.67％) with bimodal CD49d expression. Compared with patients in the CD49d negative group, patients in the CD49d positive group had higher Rai stage (*P*＝0.048) and higher proportion of spleen enlargement (*P*＝0.030). Compared with patients with homogeneous expression of CD49d, patients with bimodal expression of CD49d had a higher proportion of spleen enlargement (*P*＝0.009). The expression rate of 11q22− in bimodal CD49d^−^ group was significantly higher than that in homogeneous CD49d^−^ group (24.29％ *vs* 10.45％, *P*＝0.043). The incidence of +12 in homogeneous CD49d group was higher than that in bimodal CD49d group (16.67％ *vs* 5.95％, *P*＝0.035). The incidence of +12 in homogeneous CD49d^+^ group was higher than that in bimodal CD49d^−^ group (17.24％ *vs* 4.29％, *P*＝0.045). The incidence of +12 in homogeneous CD49d^−^ group was higher than that in bimodal CD49d^−^ group (16.42％ *vs* 4.29％, *P*＝0.024). BIRC3 mutation rate in CD49d positive group was higher than that in CD49d negative group (11.63％ *vs* 2.92％, *P*＝0.037).

**Conclusion:**

There were significant correlations between CD49d and 11q22−, +12 and BIRC3 gene mutation. Patients with bimodal CD49d were more correlated with poor prognosis indexes.

慢性淋巴细胞白血病（CLL）是一种成熟的小B淋巴细胞克隆增殖性疾病，以小B淋巴细胞在外周血、骨髓和外周淋巴器官中聚集为特点，通常发生在老年患者中，具有高度异质性[1]–[2]。CD49d是整合素家族的一种相对分子质量150×10^3^的蛋白，作为细胞间、细胞与胞外基质相互作用的黏附分子直接参与单个核细胞的迁移[3]。研究表明，CD49d表达可促进微环境介导的CLL细胞增殖，其表达水平与CLL患者的预后呈负相关[4]，是CLL的独立预后因素[5]。

既往研究报道常以CLL患者B淋巴细胞CD49d≥30％为阳性判定标准，阳性患者总生存（OS）较差[6]。临床检测显示，CD49d不仅有阴、阳性之分，还有单、双峰的表达模式。国外有学者报道了关于单、双峰差异的研究[7]，而国内尚无相关研究报道。为阐明上述两种分类方法的差异，本研究一方面将CLL患者的CD49d以阴、阳性和单、双峰分组进行两两比较；另一方面，进一步以单峰阴性（homCD49d^−^）、单峰阳性（homCD49d^+^）、双峰阴性（bimCD49d^−^）、双峰阳性（bimCD49d^+^）分组进行比较。本文首次在中国CLL患者中分析CD49d不同表达模式与分子遗传学和热点突变基因之间的关系，探索CD49d在临床预后中的价值。

## 病例与方法

1. 研究对象：纳入2017年1月至2021年6月江苏省人民医院收治的180例初诊CLL患者，收集患者的临床资料进行诊断和鉴别诊断，诊断标准参照《WHO造血与淋巴组织肿瘤分类（2016）》[8]。本研究获得了医院伦理委员会的批准（2018SRFA087），所有患者均签署知情同意书。

治疗前抽取CLL患者2 ml外周血至EDTA-K_2_抗凝管，用于流式细胞术（FCM）、荧光原位杂交（FISH）、二代测序（NGS）检测。

2. 仪器和试剂：FCM检测抗体包括：荧光标记鼠抗人单克隆抗体CD5PC7、CD19APC、CD49d-PE、CD45KO（美国Beckman公司）。FISH检测探针包括LSI 14q32探针、LSI 17p− DNA探针、LSI 11q22− DNA探针、LSI 13q14− DNA探针、LSI 6q23− DNA探针和LSI +12 DNA探针（上海基因科技公司）。DNA提取试剂盒购自北京TIANGEN公司，红细胞裂解液购自美国BD公司。

仪器包括：Beckman Navios流式细胞仪（美国Beckman公司）、ZEISS Imager.Z2荧光显微镜（德国ZEISS公司）、Illumina MiSeq测序仪（美国Illumina公司）。FISH软件为MetaSystems Isis（德国ZEISS公司），测序结果分析软件为Torrent Mapping Alignment Program（TMAP）（美国Thermo Fisher公司）。

3. FCM检测：每管分别加入混匀的100 µl抗凝外周血，加入单克隆抗体CD5PC7、CD19APC、CD49d-PE、CD45KO各5 µl。混匀，室温避光孵育15 min。加入500 µl红细胞裂解液，10 min后加入等量磷酸盐缓冲液（PBS）混匀静置5 min。1 500 r/min（离心半径10 cm）离心3 min，去上清后加入1 000 µl PBS 洗涤，最后加入500 µl PBS悬浮细胞，上机。每管至少获取500 000个细胞。先以SSC和CD45设门找出淋巴细胞，再以CD5^+^CD19^+^确定CLL肿瘤细胞，进一步分析CD49d的表达模式。以CD5^+^设门找出T淋巴细胞，以确定阴、阳性门位置。

4. FISH检测：外周血样本经低渗、固定后制片，制好的玻片在37 °C新鲜2×SSC（pH＝7.0）溶液中老化1 h，再依次置于70％、85％和100％乙醇中各2 min脱水，室温干燥。在室温下，按说明书进行LSI 14q32 DNA探针、LSI 17p− DNA探针、LSI 11q22− DNA探针、LSI 13q14− DNA探针、LSI 6q23− DNA探针和LSI +12 DNA探针的变性、杂交、洗涤、复染、镜检和信号判断。不同探针的阈值分别为14q32（8％）、17p−（5％）、11q22−（8％）、13q14−（10％）、6q23−（8％）、+12（5％）。

5. NGS检测：收集患者外周血单个核细胞1.0×10^7^个。采用DNA提取试剂盒提取基因组DNA，以至少100 ng DNA进行扩增建库，应用Illumina MiSeq测序仪对基因外显子热点区域测序，平均测序深度1000×，使用TMAP软件分析测序结果。6个热点突变基因包括：TP53、ATM、NOTCH1、SF3B1、BIRC3、MYD88。

6. 统计学处理：实验数据采用SPSS 20.0软件进行统计分析，计数资料以例数（百分比）表示，计量资料用平均数（范围）表示。率的比较采用卡方检验，*P*<0.05为差异有统计学意义。

## 结果

1. CD49d表达模式：对180例CLL患者外周血标本进行CD49d表达分析。以CD49d≥30％为阳性判定标准，CD49阴性患者137例（76.11％），CD49d阳性患者43例（23.89％）。96例（53.33％）患者CD49d表达均一，表达模式为单峰；84例（46.67％）患者CD49d有两个独立的细胞群，表达模式为双峰。结合两种分组依据，进一步将患者分为homCD49d^−^、homCD49d^+^、bimCD49d^−^、bimCD49d^+^四组。

2. CD49d表达模式与CLL患者临床特征的相关性：180例CLL患者中，男116例，女64例，平均年龄61（33～89）岁。以CD49d表达模式为分组依据，进行患者临床特征的比较。与CD49d阴性组患者相比，CD49d阳性组患者Rai分期高（*P*＝0.048），脾脏增大比例高（*P*＝0.030）。与CD49d单峰患者相比，CD49d双峰患者脾脏增大比例高（*P*＝0.009）（表1）。

**表1:** 180例慢性淋巴细胞白血病（CLL）患者CD49d表达模式与临床特征的相关性［例（％）］

基本特征	CD49d阴性与阳性				CD49d单峰与双峰			
	CD49d阴性（137例）	CD49d阳性（43例）	*χ*^2^值	*P*值	CD49d单峰（96例）	CD49d双峰（84例）	*χ*^2^值	*P*值
年龄			1.462	0.294			1.528	0.234
≤60岁	75（41.67）	19（10.55）			46（25.56）	48（26.66）		
>60岁	62（34.44）	24（13.34）			50（27.78）	36（20.00）		
性别			0.011	1.000			3.665	0.062
男	88（48.89）	28（15.55）			68（37.78）	48（26.66）		
女	49（27.22）	15（8.34）			28（15.56）	36（20.00）		
Rai分期			9.599	0.048			6.105	0.192
0期	11（6.11）	0（0）			5（2.78）	6（3.33）		
Ⅰ期	21（11.67）	5（2.77）			9（5.00）	17（9.44）		
Ⅱ期	26（14.44）	3（1.67）			14（7.78）	15（8.33）		
Ⅲ期	25（13.89）	11（6.11）			22（12.22）	14（7.78）		
Ⅳ期	54（30.00）	24（13.34）			46（25.56）	32（17.78）		
Binet分期			1.772	0.412			3.214	0.201
A期	21（11.67）	9（5.00）			14（7.78）	16（8.89）		
B期	49（27.22）	11（6.11）			28（15.55）	32（17.78）		
C期	67（37.22）	23（12.78）			54（30.00）	36（20.00）		
CLL国际预后指数			0.175	0.916			0.113	0.945
0～3分	60（33.33）	18（10.00）			41（22.78）	37（20.55）		
3～7分	59（32.78）	20（11.11）			42（23.33）	37（20.56）		
8～10分	18（10.00）	5（2.78）			13（7.22）	10（5.56）		
风险等级			5.056	0.168			0.712	0.634
低	26（14.45）	4（2.22）			13（7.22）	17（9.44）		
中	34（18.88）	15（8.34）			28（15.55）	21（11.67）		
高	49（27.23）	19（10.55）			38（21.11）	30（16.67）		
极高	28（15.55）	5（2.78）			17（9.44）	16（8.89）		
脾脏增大			5.069	0.030			7.243	0.009
是	79（43.89）	33（18.33）			51（28.33）	61（33.89）		
否	58（32.22）	10（5.56）			45（25.00）	23（12.78）		
复发			0.034	1.000			2.210	0.177
是	37（20.56）	11（6.11）			30（16.67）	18（10.00）		
否	100（55.55）	32（17.78）			66（36.66）	66（36.67）		
Richter转化							0.218	1.000
是	3（1.67）	0（0）	0.958	1.000	2（1.11）	1（0.56）		
否	134（74.44）	43（23.89）			94（52.22）	83（46.11）		
生存状态			0.369	1.000			1.794	0.254
死亡	6（3.33）	1（0.56）			2（1.11）	5（2.78）		
存活	131（72.78）	42（23.33）			94（52.22）	79（43.89）		

3. CD49d与分子遗传学及突变基因的关系：按照CD49d的不同表达模式进行分组，比较各组分子遗传学异常14q32、17p−、11q22−、13q14−、6q23−、+12及TP53、ATM、NOTCH1、SF3B1、BIRC3、MYD88基因突变情况（表2）。TP53、ATM、NOTCH1、SF3B1、BIRC3、MYD88基因的突变率分别为17.78％、17.22％、14.44％、10.56％、5.00％、13.89％。

**表2:** 180例慢性淋巴细胞白血病患者CD49d表达模式与分子遗传学及基因突变的相关性［例（％）］

检测项目	CD49d单峰阴性（67例）	CD49d单峰阳性（29例）	CD49d双峰阴性（70例）	CD49d双峰阳性（14例）	*P*_1_	*P*_2_	*P*_3_	*P*_4_	*P*_5_	*P*_6_	*P*_7_
分子遗传学异常											
14q32	13（19.40）	5（17.24）	6（8.57）	2（14.29）	0.804	0.092	1.000	0.615	0.291	0.085	1.000
17p−	13（19.40）	5（17.24）	12（17.14）	0（0）	0.358	0.548	1.000	0.203	1.000	0.826	0.156
11q22−	7（10.45）	6（20.69）	17（24.29）	4（28.57）	0.503	0.058	0.203	0.742	0.798	0.043	0.704
13q14−	24（35.82）	8（27.59）	31（44.29）	5（35.71）	0.282	0.219	0.487	0.768	0.175	0.384	0.726
6q23−	0（0）	1（3.45）	3（4.29）	1（7.14）	0.594	0.186	0.302	0.525	1.000	0.245	1.000
+12^a^	11（16.42）	5（17.24）	3（4.29）	2（14.29）	0.284	0.035	1.000	0.192	0.045	0.024	1.000
基因突变											
TP53	11（16.42）	5（17.24）	16（22.86）	0（0）	0.262	0.700	1.000	0.062	0.601	0.394	0.156
ATM	10（14.93）	6（20.69）	11（15.71）	4（28.57）	0.250	0.846	0.555	0.264	0.567	1.000	0.704
NOTCH1	12（17.91）	6（20.69）	6（8.57）	2（14.29）	0.455	0.092	0.780	0.615	0.104	0.132	1.000
SF3B1	5（7.46）	3（10.34）	10（14.29）	1（7.14）	1.000	0.338	0.694	0.681	0.750	0.276	1.000
BIRC3	3（4.48）	3（10.34）	1（1.43）	2（14.29）	0.037	0.506	0.362	0.071	0.074	0.359	1.000
MYD88	9（4.48）	3（10.34）	11（15.71）	2（14.29）	0.802	0.667	1.000	1.000	0.752	0.810	1.000

注：^a^：仅伴+12。*P*_1_：阴性组与阳性组比较；*P*_2_：单峰组与双峰组比较；*P*_3_：CD49d单峰阴性组与CD49d单峰阳性组比较；*P*_4_：CD49d双峰阴性组与CD49d双峰阳性组比较；*P*_5_：CD49d单峰阳性组与CD49d双峰阴性组比较；*P*_6_：CD49d单峰阴性组与CD49d双峰阴性组比较；*P*_7_：CD49d单峰阳性组与CD49d双峰阳性组比较

结果显示，bimCD49d^−^组11q22−突变率高于homCD49d^−^组（*P*＝0.043）。在+12突变率比较中，CD49d单峰组高于双峰组（*P*＝0.035），homCD49d^+^组高于bimCD49d^−^组（*P*＝0.045），homCD49d^−^组高于bimCD49d^−^组（*P*＝0.024）。CD49d阳性组BIRC3基因突变率高于CD49d阴性组（*P*＝0.037）。

## 讨论

CD49d是CD49d/CD29整合素异二聚体非常晚期抗原4（VLA-4）的α链，作为CLL的独立预后指标之一而备受关注[9]。既往的研究报道通常以CD49d的阴、阳性作为判断标准。近期发现，CD49d存在单、双峰表达模式，预后情况也存在差异[7]。本研究首次将CD49d阴、阳性和单、双峰表达模式结合，在中国人群中探索CD49d的不同表达模式与分子遗传学和热点突变基因的关系。

我们发现，与CD49d阴性患者相比，阳性患者Rai分期更高、更易脾肿大；与CD49d单峰患者相比，双峰患者更易脾肿大。CD49d阳性与预后的关系已有报道[4]，在本研究中得到进一步证实。肿瘤细胞可迁移至髓外器官，如中枢神经系统、脾脏、肝脏等，造成疾病进展。CD49d有介导黏附和迁移作用[10]，CD49d阳性组和双峰组更易脾肿大表明其肿瘤细胞迁移能力更强，临床表现更差。

此外，我们发现CD49d表达模式与分子遗传学指标11q22−、+12存在显著相关性。ATM基因位于人类染色体11q22-23，主要参与细胞周期、DNA损伤修复和凋亡等一系列重要细胞功能的信号传导，起到枢纽作用。ATM基因缺陷患者患血液病的风险是正常人的数百倍，且有超过20％的患者被诊断为CLL[11]，在预后风险等级中为高风险基因。本研究结果显示，bimCD49d^−^组11q22−发生率显著高于homCD49d^−^组。+12是CLL中常见的染色体异常，发生于15％～20％的病例，在60％的病例中是唯一的细胞遗传学改变。在临床常用的遗传学预后分层指标中，仅出现+12的CLL患者预后较好。少数情况下，+12伴IgHV、NOTCH1等基因突变是预后不良的标志[12]–[13]。本研究发现CD49d单峰的+12突变率明显高于双峰表达模式；homCD49d^−^组的+12突变率显著高于bimCD49d^−^组。11q22−与+12的结果说明，CD49d单峰与双峰表达模式存在差异，且双峰与预后不良指标更相关。此外，研究提示homCD49d^+^组的+12突变率高于bimCD49d^−^组。因此，我们认为，传统方法仅采用CD49d≥30％为划分依据并不完善，CD49d阴、阳性结合单、双峰表达模式较传统判断方法更为客观。

近年来，DNA测序技术一直是分子生物学相关研究中最常用的技术手段之一。Illumina测序仪使用的方法是克隆单分子阵列技术，具有高通量、速度快的优点，在疾病的诊断、精准治疗和预后判断中具有不可替代的作用。我们研究了CD49d的表达模式与CLL 6种热点突变基因TP53、ATM、NOTCH1、SF3B1、BIRC3、MYD88的关系。结果显示，CD49d的表达特点与BIRC3具有显著相关性。BIRC3是NF-κB信号的中央调节器，在缺乏微环境信号的情况下起到通路抑制作用[14]。BIRC3作为CLL的热点突变基因，与11q22−和TP53相关，BIRC3突变与CLL进展风险密切相关，阳性患者预后更差[15]–[16]。在本研究中，CD49d阳性组的BIRC3突变率显著高于阴性组，与文献报道相符。CD49d作为独立预后因素，理论上与CLL国际预后指数（CLL-IPI）中的重要参数TP53突变相关，但在本研究中未发现差异有统计学意义，可能与TP53突变阳性率较低[17]、研究纳入的样本数较少有关。

根据以上研究结果，CD49d不仅与Rai分期、脾肿大等临床特征相关，也与11q22−、+12和BIRC3基因突变相关。一方面，CD49d阴、阳性与分子遗传学和热点突变基因的关系与文献报道相符，阳性患者与预后较差指标更相关；另一方面，CD49d单、双峰间亦存在明显差异，CD49d双峰与预后较差指标更相关。本文首次提出了bimCD49d^−^患者的临床意义，将其单独划分，对既往文献进行了有效的补充。因此，我们认为，CD49d单、双峰表达模式的预后价值值得进一步研究。今后，我们将进一步扩大样本量，进行治疗前后CD49d动态监测，总结CD49d与患者治疗方案、预后等的关系，进一步挖掘该分子的临床预后价值。
